# Information Security in Medical Robotics: A Survey on the Level of Training, Awareness and Use of the Physiotherapist

**DOI:** 10.3390/healthcare10010159

**Published:** 2022-01-14

**Authors:** Lisa Monoscalco, Rossella Simeoni, Giovanni Maccioni, Daniele Giansanti

**Affiliations:** 1 Faculty of Engineering, Tor Vergata University, Via Cracovia, 00133 Rome, Italy; lisamonoscalco@hotmail.com; 2 Facoltà di Medicina e Chirurgia, Università Cattolica del Sacro Cuore, Largo Francesco Vito, 1, 00168 Rome, Italy; rossella.simeoni.1955@gmail.com; 3 Centre Tisp, Istituto Superiore di Sanità, 00161 Rome, Italy; giovanni.maccioni@iss.it

**Keywords:** medical devices, rehabilitation, assistance, robotics, cyber security

## Abstract

Cybersecurity is becoming an increasingly important aspect to investigate for the adoption and use of care robots, in term of both patients’ safety, and the availability, integrity and privacy of their data. This study focuses on opinions about cybersecurity relevance and related skills for physiotherapists involved in rehabilitation and assistance thanks to the aid of robotics. The goal was to investigate the awareness among insiders about some facets of cybersecurity concerning human–robot interactions. We designed an electronic questionnaire and submitted it to a relevant sample of physiotherapists. The questionnaire allowed us to collect data related to: (i) use of robots and its relationship with cybersecurity in the context of physiotherapy; (ii) training in cybersecurity and robotics for the insiders; (iii) insiders’ self-assessment on cybersecurity and robotics in some usage scenarios, and (iv) their experiences of cyber-attacks in this area and proposals for improvement. Besides contributing some specific statistics, the study highlights the importance of both acculturation processes in this field and monitoring initiatives based on surveys. The study exposes direct suggestions for continuation of these types of investigations in the context of scientific societies operating in the rehabilitation and assistance robotics. The study also shows the need to stimulate similar initiatives in other sectors of medical robotics (robotic surgery, care and socially assistive robots, rehabilitation systems, training for health and care workers) involving insiders.

## 1. Introduction

Cybersecurity (*Cyb*) in healthcare (*CybH*) includes all the general actions that we can find in the world of industry and consumption (*network security, application security, information security, operational security, disaster recovery and operational continuity, end-user training*), adjusted specifically for the *health domain* [1,2].

*CybH* addresses the cyber risk in a *cyber-system* in the *health domain*. The *cyber-system* can either be a complex medical device and/or a complex interoperable and heterogeneous system (e.g., a hospital information system, a radiology information system; a dedicated medical network). Important issues emerge for medical devices (MDs).

In the case of a *standalone medical device (SMD)* (not connected to other systems) *CybH* must concentrate on the device itself. Much of the Cyb depends on the correct implementation of the certification processes, considering also the CybH.

If the device *is not standalone, i.e., it is an interconnected Medical Device* (IMD), in addition to a certification process, it is also necessary to consider the *Cyb* vulnerability of the IT environment (e.g., hospital information system, the network of the rehabilitation centre, the home WI-Fi).

*Nowadays, it is rare to find SMDs. Most MDs are IMDs*. Examples are the *artificial pancreas* and the *pacemaker*. They need a communication link to an IT environment, both for the monitoring and/or updating functions [3,4,5,6].

*Medical robots used in rehabilitation and assistance* [7,8] *are examples of IMDs: they need a communication link to exchange and record data, for updating and and/or other functions.*

### 1.1. Regulatory and Legislative Issues in Medical Robotics

Safety and security concepts are at the base of the *Cyb* of rehabilitation and assistance robots.

In general, when we talk about safety we must distinguish well between safety and security [9]. The term “safety” concerns protections and countermeasures against actions, conditions or circumstances that could harm (physically and/or psychologically) living beings, and particularly humans (see for example the IETF Internet Security Glossary [10]). The term “security” is sometimes used as a broader term encompassing “safety”; however, it is more often used in relation to assets more diverse than living beings, such as data, networks, computers, and money. In the context of cyber-physical systems, the term usually refers to data, hardware, or computing processes. The typical case of using the robot is as an IMD in the hospital (or similar facility) or at home. Therefore, regarding IMD robot safety and security, the medical device itself, the environments of use (for example, the hospital or the home), and the organization and working regulations must be taken into consideration.

The problem is very broad and includes: (a) the safety of the patient and the worker (e.g., the physiotherapist); (b) the regulations for the medical devices; (c) the regulations for the safe use of networks; and (d) other interrelated regulations, such as product safety in general or radio directives. Both work safety and patient safety in Europe present a very complex regulation framework. In any case, the employer/hospital manager is always responsible for both safety and security (from delinquent actions) and this applies also to *cyber-systems*.

The European Union has recently recalled the entire existing regulation framework [11] through a Communication from the Commission to the European Parliament, the Council, the European Economic and Social Committee and the Committee of Regions. This Communication regards the practical implementation of the provisions of the Health and Safety at Work Directives [11]. In [12], an examination of the European regulations on patient safety and, more generally, hospital safety is reported.

Fosch-Villaronga and Mahler provided in their recent study [13] a very fine analysis in this direction, for the European framework, identifying problems and criticisms with regard to points (b) to (d) above. *As a first step,* they considered the relationship between robots in the *health domain* and the European general product safety regulations (*Directive 2001/95/EC of the European Parliament and of the Council of 3 December 2001 on general product safety 2001, and Directive 85/374/EEC on liability for defective products*) [14].

They highlighted that the applicability of product liability laws is not straightforward for the robots, comprising cyber-physical systems.

*As a second step*, they analyzed the impact of the medical device regulation (MDR) (Regulation (EU) 2017/745) [15] on the robots.

*Finally*, they focused on the three legal frameworks partially regulating robot *Cyb* (NIS Directive, GDPR, Cybersecurity ACT) [16,17,18] both as MD and IMD interconnected to a network. The scholars reported that also other regulations impacted on *Cyb*. They gave the example of the EU Radio Equipment Directive [19].

The authors highlighted [13] the novelty of the MDR. They also highlighted the *shadows*. The *first shadow* is that MDR focuses heavily on manufacturers and little on recipients/users. The *second* *shadow* is that compliance with cybersecurity requirements is challenging, due to the potential overlap of different certification schemes (with varying geographical or product scope) and to the evolution of regulations external to the MDR [14].

The *third shadow* is that the specific *Cyb* certifications are voluntary, as in the case of the *cybersecurity ACT* [18]. We found another important *shadow*. The intended use and certification as MD do not always seem aligned (for example when MDs used in rehabilitation are not certified for this) [20]. *Cyber-attacks can have serious physical and/or psychological impacts* [12], as described by means of a model in [13].

### 1.2. The Medical Robots Used in Rehabilitation and Assistance and Cybersecurity

An important sector for medical robots is that of rehabilitation and assistance.

Robotics in rehabilitation [7,21,22,23,24,25,26,27,28,29,30,31] essentially concerns three sectors:

- Balance (BA)
- The lower limbs (LOLI)
- The upper limbs (UPLI)

These sectors use *exoskeleton* or *end-effector technology*. The exoskeletal robot completely covers the limb, following and replicating the human anthropometry. The mechanics guide each segment involved in the rehabilitation practice. Therefore, an exoskeleton is a “mechatronic” apparatus. It is worn and performs the same kinematic/dynamic activity practiced by the patient. In a robotic end-effector device, the input for carrying out the rehabilitation exercise comes directly from the distal part of the limb. It allows the natural kinematic activation of the movement, without unnatural constraints.

*Assistance robotics* uses ”social robots” (SRs) [8,32]. Use of these devices has recently increased, to overcome the problem of social distancing in the Covid-19 pandemic.

Today, SRs are designed to:

- Interact with people, even by touching them, since the physical contact helps to establish a better emotional relationship.
- Assist people with many daily activities (as a reminder or as a kind of butler).
- Assist people in medical activities, such as drug administration and patient monitoring.
- Support physicians in physical rehabilitation, such as *Pepper*, which supports physiotherapists during sessions [33,34,35,36], or support patients in their movements or displacements (e.g., *Robear* [37,38] transports patients).
- Support people with complex communication needs.
- Support families or therapists as cultural mediators.

The SRs are a totally new challenge for *CybH*. There are important aspects related to *Cyb* that require consideration in these devices, since their programming has important implications for the robot’s moral behaviour, resulting in the interdisciplinary field of machine ethics [39,40,41,42,43,44,45]—that is, how to program robots with ethical rules [40].

**Figure 1:**
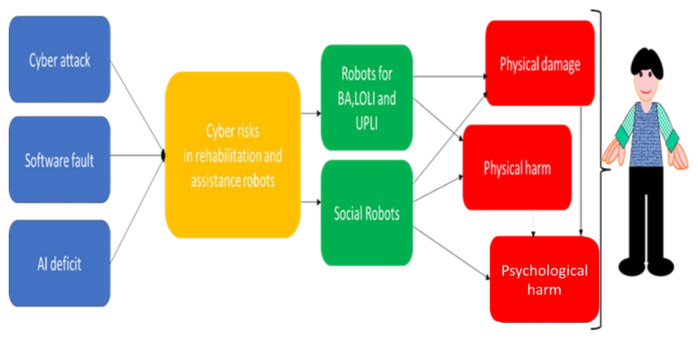
Model of the impact of the cyber-attacks in the investigated field.

This sector involves “adding an ethical dimension to the machine” [45], and it has become of utmost importance because of wonderful technological developments in the field of the CRs and, more generally, artificial intelligence [41,42,43,44,45]. Gordon [39] highlighted that making ethics “computable” depends in part on how the designers understand ethics and attempt to implement that understanding in programs, but also on their expertise in the field of human–robot interaction. He found that researchers and programmers have neither a good enough understanding nor sufficient ethical expertise to build moral machines that would be comparable to human beings with respect to ethical reasoning and decision-making. Figure 1 shows the modelling of the physical and psychological impact [13], developed by us for the rehabilitation and assistance robotics. Note that psychological harm can also occur as an indirect consequence of physical damage or harm caused by rehabilitation robots. It is therefore clear that there is a strong need for studies to help develop consensus in this area. It is important to stimulate the stakeholders to face these problems. It is also important to sensitize scholars to invest energies in research initiatives.

### 1.3. Motivation and Purpose of the Study

It is vital to plan an acculturalization process on *Cyb*. This process must concern all the actors involved, from the builders up to the users and the caregivers, in the different environments (from home up to the hospital).

Training in this area must also become an important issue. Stakeholders will have to start specific monitoring initiatives, through targeted surveys, for example, to verify the state of diffusion of the *Cyb* culture in robotics, and assess the consensus and opinion in this area. This is an important and preliminary step in the launch of agreements and consensus initiatives for these devices, also considering that *Cyb* certification of CRs is voluntary. At present, there are no active initiatives of this type. A search on Pubmed with the key *“cyber security” [Title/Abstract] AND “robotics” [Title/Abstract] AND “questionnaire” [Title/Abstract]* (also trying with synonyms) did not show results.

In other sectors of the *health domain*, where technology is rapidly developing, ad hoc questionnaires have been developed with the aim of investigating the consensus between the actors. For example, in digital radiology, various studies have focused on different actors and conducted research through questionnaires on a very important issue relating to *information technology in cyber-systems*, that of *artificial intelligence*. Selected papers [46,47,48,49,50,51,52,53,54,55,56] highlight studies focused on some of the actors concerned: radiologists and radiographers [49,50,51,52,53,54], primary care providers [51], students [55], and patients [46,47,48], that is, both on service providers and users, and on the subjects in training. The importance of training and the usefulness of free questionnaires emerged from these studies. Surveys were used both to collect interviews and structured data from focus groups/consensus initiatives. In all cases identified, original questionnaires based on choice questions Likert scales, graded questions (in a psychometric scale) and open-ended questions were used. With very few exceptions [48], scholars preferred to use personal and original rather than validated/standardized questionnaires to investigate the topic.

For this reason, we consider a similar approach as regards robot technology (also rapidly evolving) to be useful on another topic connected to *information technology in cyber-systems*, that of *Cyb*, where, similarly, training plays a leading role. For this reason, we believe it is equally useful to propose it to the professionals involved in this area.

Many professionals in the *health domain* have to do with the robots in rehabilitation and assistance (from the bioengineer up to the physiotherapist). The physiotherapists are key professionals in this field. It is therefore important to investigate the relationship between the physiotherapist and *CybH*.

This is useful to provide *medical knowledge* and stimulate stakeholders to recommend initiatives.

We have therefore set ourselves the goal to focus on the physiotherapist and: (1) to investigate the consensus, familiarity, and opinion on *Cyb* in this field, based both on the training and experience in the *workplace*; (2) to apply an electronic questionnaire designed for the investigation.

## 2. Materials and Methods

In line with the aim of the study, we decided to develop an electronic questionnaire to investigate the acceptance and the consensus of the physiotherapists. We used Microsoft Forms (Microsoft Corporation, Albuquerque, Nuovo Mexico (NM), USA), available in the Microsoft 365 App Business Premium suite in the *workplace*. It is the software product recommended by the company’s Data Protection Office (DPO). It is included in the informatic domain and complies to the regulations on data privacy and security. We adhered to the *SURGE Checklist* [57] for the development and administration of the questionnaire. The questionnaire used different type of questions: *open questions, choice questions, multiple choice questions, Likert scales, graded questions*. A six-level psychometric scale was used both in the graded questions and in the Likerts. Therefore, it was possible to assign a minimum score of one and a maximum score of six. The theoretical mean value (TMV) was equal to 3.5. We used the TMV for comparison in the analysis: an average value below the TMV shows a more negative than positive response, whereas an average value above TMV indicates a more positive than negative response.

For the check of data normality, we used the Kolmogorov–Smirnov test, which is preferable for sample sizes like ours. The software SPSS V. 25.0 (IBM SPSS software, Armonk, NY, USA) was used in the study. The Cohen’s d effect size estimated with 0.499 the effect size. A sample with *n* > 60 was estimated to be suitable for the study. We submitted the survey from 1 June 2021 until 20 October 2021.

We have submitted the questionnaire to the physiotherapists using social networks, web sources, messengers, and lists/webs from professional associations.

**Figure 2:**
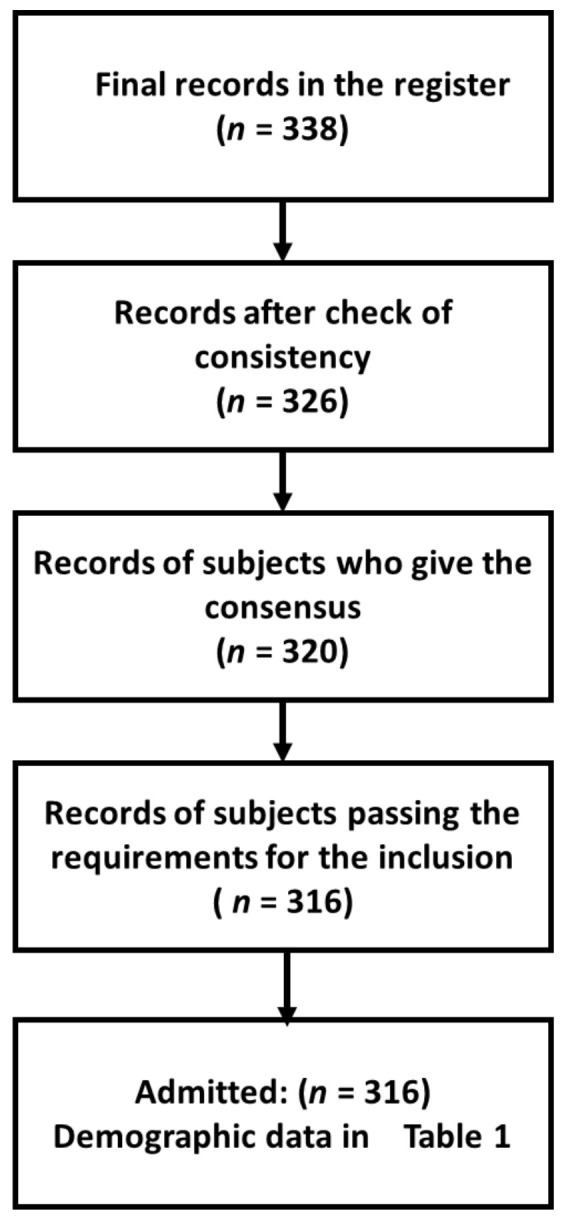
Diagram describing the inclusion process.

Figure 2 reports the diagram of the inclusion process. Table 1 shows the demographic characteristics.

The methodology, based on an electronic survey, focused on the physiotherapist. It investigated, through the tools available in the survey, the different aspects of *Cyb*.

The electronic survey is arranged into five sections (see Table 2).

*Section 1* is designed for collecting the demographic data (reported in Table 1). *Section 2* investigates if there is an interaction with the robots in the workplace and whether this interaction also concerns *Cyb*. *Section 3* investigates the specific training on *Cyb* and on the connected disciplines. *Section 4* proposes *self-assessment* questions regarding *Cyb* while interacting with the robots. *Section 5* collects both proposals and the cyber-risk experiences in one’s work environment useful both for the reader and the stakeholder.

## 3. Results

The results are reported in the four sections below. For each section, the type of questions, the questions asked, and the statistics are reported.

### 3.1. Output from Section 2 “Robotics and Cybersecurity in the Workplace”

As a first aspect, we investigated the use of rehabilitation robotics and the involvement (role) of physiotherapists in its use, either as active users or just observers. A *multiple-choice* question was proposed (relating to three different robots used in rehabilitation).

**Figure 3:**
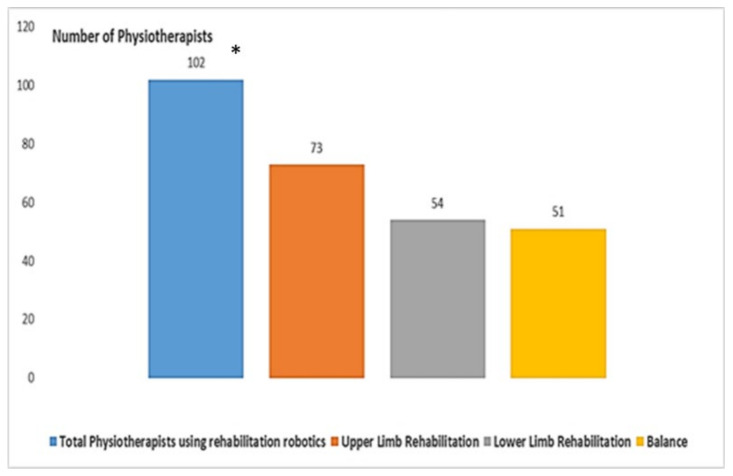
Use of rehabilitation robotics in the workplace (* 102 is different from the sum of the three choices, because it is a multiple-choice question).

Figure 3 shows that only 102 (32.27%) respondents use rehabilitation robotics in the *workplace*. In detail, 73 (23.10%) use robotics in upper limb rehabilitation. A smaller number use robotics in the other two sectors of balance (54, 17.08%) and lower limb rehabilitation (51, 16.14%).

A second question with two choices (*Yes/No*) also investigated involvement in *Cyb* activity.

**Figure 4:**
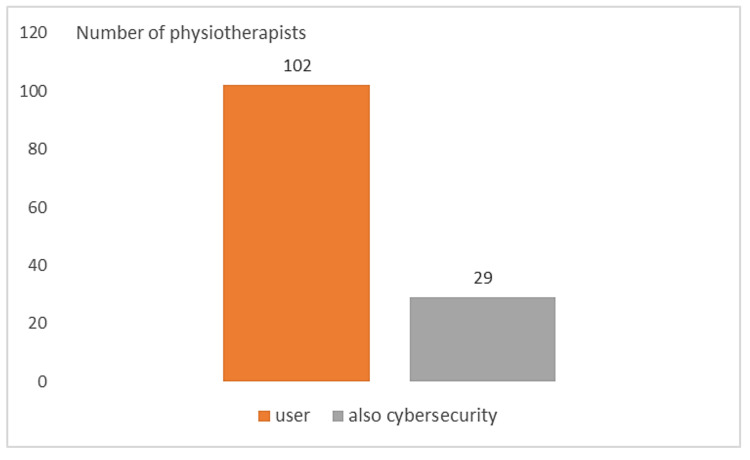
Role of the use of rehabilitation robotics by physiotherapists.

Figure 4 highlights that all the interviewed people reported the role of technology user. Only 29 (9.18%) claimed to have been involved in the *CybH*, resulting in a significantly low number (*p*-Value < 0.01, χ2test).

**Figure 5:**
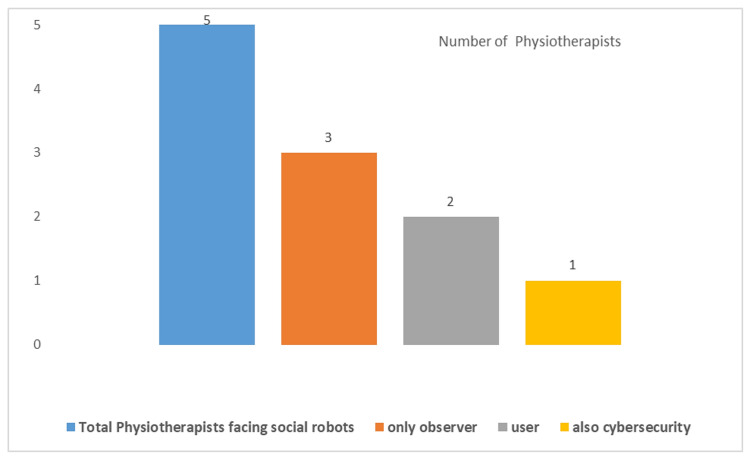
Physiotherapists’ interaction with social robots.

It is well known that the use of SR is still very limited. However, we wanted to investigate any involvement, which could also concern research projects. Three questions were proposed. A question with two choices (*Yes/No*) investigated the SR presence in the *workplace*. A question with two choices (*only observer/user*) investigated the role in the interaction. A question on their role in *Cyb* was also proposed to those who had responded “user”. Figure 5 highlights that only 5 respondents stated that they were dealing with SRs. Three (0.95%) declared that they were observers, two (0.63%) were users, and only one (0.32%) faced *CybH issues*. These frequencies also had a high statistical significance (*p-*value < 0.01, χ2test).

### 3.2. Output from Section 3 “Training in Cybersecurity and Robotics”

Table 3 reports *the perceived level of training on SRs,* *robots for BA, robots for LOLI, robots for UPLI*. Four graded questions with 6 levels of score (1 = min; 6 = max) were used.

The most popular response was *Robots* for UPLI. The least popular answer was *Social Robots*. All the answers received a score above the TMV.

Table 4 reports the *Perceived training on informatics, mHealth, eHealth, cybersecurity*. Four graded questions with 6 levels of score (1 = min; 6 = max) were used.

The most popular response was *informatics*. The least popular answer was *Cyb*. All the answers obtained a score above the TMV except for *Cyb*.

Table 5 reports the perceived training on *Cyb* with reference to the different cyber-attacks. A Likert scale was used with the modules associated to each cyber-attack. Each module had 6 levels (1 = min; 6 = max). Results show low scores, all below the TMV, except for *malware, phishing, and password crackers* (just above the threshold).

We asked also to indicate (*based on the training) the sector mostly affected by the problem of Cyb*. A Likert scale was used with the modules associated to each robot. Each module had 6 levels (1 = min; 6 = max). Table 6 reports the responses related to the specific Likert scale. The most popular response was the SR. The least popular answer was the BA. All the answers received a score above the TMV.

We completed this section asking specific further questions on the regulatory issues and on the awareness of the role with *Cyb*. Two graded questions with 6 levels of score (1 = min; 6 = max) were used for investigating the training on regulatory issues. The first question investigated the training on the regulatory issues on *Cyb*. The second question investigated the training on the regulatory issues on *Cyb*, specifically referring to robotics.

**Figure 6:**
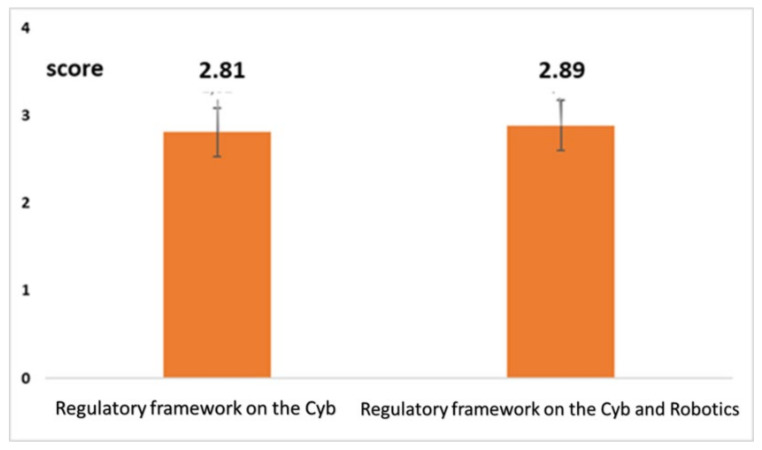
Level of training on the regulatory framework (also referred to robotics).

**Figure 7:**
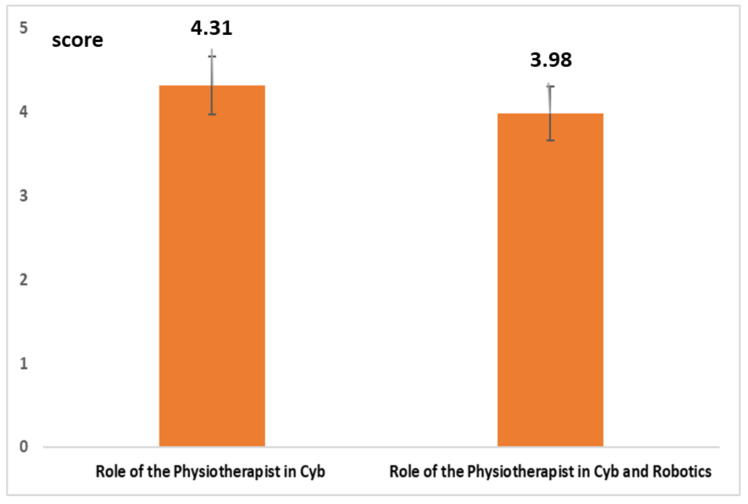
Level of awareness on the role of the physiotherapist on Cyb.

Figure 6 highlights a very low level of training on regulatory issues both as a whole (average value = 2.89; confidence interval (CI) 95%: ±0.35) and related to robotics (average value = 2.88; CI 95%: ±0.35). Two graded questions with 6 levels of score (1 = min; 6 = max) were used for investigating awareness on their role with *Cyb*. The first question investigated the awareness of the role with *Cyb*. The second question investigated awareness of the role with *Cyb* and robotics. Figure 7 highlights a level of awareness well above the TMV (with reference to the role of the physiotherapist in *Cyb* as a whole (average value = 4.31; CI 95%: ±0.38) and while interacting with robotics (average value = 3.98; CI 95%: ±0.37).

### 3.3. Output from Section 4 “Self-Assessment on Cybersecurity and Robotics”

This section considers the self-assessment scenarios of familiarity with *Cyb*. *A first investigation* involved a mapping of cyber-attacks in relation to the four robots (Table 7). Each one of the cyber-attacks was proposed with *multiple choices* (LOLI, UPLI, BA, SR). The interviewees could indicate the applicability or non-applicability of cyber-attacks with the robots. Table 6 highlights how *malware, phishing and password crackers* were the most indicated. However, a statistical frequency analysis did not show significance (χ2test, *p*-Value = 0.221).

*A second investigation* (Table 8) concerned the model proposed in Figure 1. The functional problems (*physical damage, physical harm, physiological harm*) were proposed with *multiple choices* (LOLI, UPLI, BA, SR). The SRs showed the lowest scores for *physical harm*, with statistical significance (χ2test, *p*-Value = 0.048) and physical damage with statistical significance (χ2test, *p*-Value = 0.049). However, the SRs showed the highest score for *psychological harm*, with a high statistical significance (χ2test, *p*-Value = 0.008).

As *a third investigation* we proposed a specific risk self-assessment (Table 9, Table 10, Table 11 and Table 12). A Likert scale was proposed for each one of the robots (UPLI, LOLI, BA, SR). The modules in the Likert were identical. Each module had 6 levels of score (1 = min; 6 = max). The scores almost overlapped and were above the TMV for UPLI, LOLI, BA. For these robots the scenario “On the possible effect on the patient/practitioner’s health and safety” obtained the highest score. All the values were below the threshold for the SRs, except for the score associated with the scenario “On the possible effect on the patient /practitioner’s health and safety”.

### 3.4. Output from Section 5 “Proposals and Collection of Personal Experiences of Cyber-Risk”

As *a final investigation* we have invited respondents to: (a) freely express opinions and suggestions on cyber-risks and actions to consider shortly; (b) cite personal experiences related to *Cyb* problems. Open-ended questions were used in this section.

#### 3.4.1. Proposals

We grouped and categorized similar questions. Table 13 reports the suggestions for the most probable cyber risks to face. The most worrying concern was the *physical damage* caused by an incorrect imposition of motion. Table 14 reports the suggestions related to the actions to consider. The most suggested action was related to the periodic monitoring activities managed by the scientific societies.

#### 3.4.2. Collection of Personal Experiences of Cyber-Risk

We also invited the physiotherapists to describe an experience in this field. There was an open space of about a half page of space for this. Both the participants with a direct experience on robotics and the participants with only a training experience contributed with enthusiasm. 302 (95.57%) physiotherapists described an experience of a problem with *Cyb* in the workplace. 55 participants reported *Cyb* problems with robotics in the workplace. We should consider that in *Section 2* (see Section 3.1) it emerged that 102 physiotherapists work with rehabilitative robotics and 2 deal with SRs as users. This means that 52.3% of them were involved in a *Cyb* problem.

**Figure 8:**
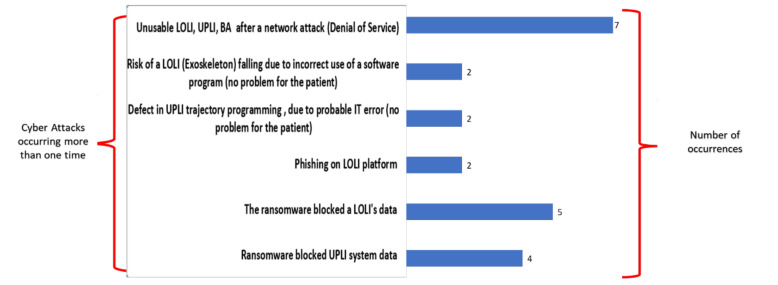
Experiences with cyber-attacks which occurred more than one time after categorization.

The problems have been analyzed and categorized. The problems that occurred more than one time are shown in Figure 8. Figure 8 highlights how the two most frequent reported and described attacks were the *denial of service* (7 times), which involved a network with LOLI, UPLI, BA, and ransomware attacks on the data of a LOLI platform (5 times).

## 4. Discussion

Mechatronic devices have grown in importance in recent years [7,24]. Among these devices we certainly find the robots for rehabilitation and assistance [8,21,22,23,24,25,26,27,28,29,30,31,32]. The increased use of these technologies raises important issues on *Cyb*. It is important to investigate the perceptions of the insiders, also in robotics, as for other disruptive technologies [58].

We started with the physiotherapist, who is facing a transformation towards digitalization in the pandemic era, as has been highlighted by A. Lee in [59].

In this study we have proposed a useful electronic questionnaire. It included: *open-ended questions, choice questions, multiple choice questions, Likert scales, and graded questions*. It permitted collection of important data on: *(a) the use of robotics and direct involvement in the CybH; (b) training in robotics, cybersecurity, and other disciplines; (c) self-perception of* *cybersecurity and robotics; (d) opinions, suggestions, and experiences*.

When we place our investigation in the international context, we must consider the following. *Cyb* has vast implications in the *health domain* and it is evident that it has been the subject of many targeted studies [60]. However, the number of the studies focusing also on robotics is extremely low [61]. The research [60] in Pubmed (the most important database of the *health domain*) shows that, to date, no one has yet addressed specific issues of *Cyb* in robotics, submitting questionnaires to medical professionals.

The questionnaire, dedicated to physiotherapists and with reference to *CybH* in robotics, has the advantage of allowing the monitoring of roles and interactions in the workplace, monitoring of training received, a self-assessment of risks, and a virtual focus group.

The study has some limitations. *A first limitation* is that the questionnaire is both dedicated to one field of the medical robotics (the rehabilitation and assistance robotics) and calibrated on a professional group. Many professional groups play an important role in rehabilitation and assistance robotics. Specialized questionnaires for these professional groups should be developed in the future.

*Another second limitation* is the limitlessness of the theme. It is impossible to address all the implications in a single study.

In particular, the ethical implications of robotics are very important. These implications will have a strong impact on *Cyb* and require a very robust and multidisciplinary approach involving all the actors.

There are two important macro-sectors of ethics with an impact on *Cyb*. The first macro-sector is the ethics in a responsible research and innovation [62]. Stahl and Coeckelbergh highlighted, for the first macro-sector, the important implications of *Cyb* [63,64,65,66,67,68,69,70] in the replacement of the human in work, as regards the responsibility for and in the management of information. The second macro-sector is the ethics problem encountered while building moral robots [39]. This focuses on the interdisciplinary field of machine ethics.

The *third limitation* is that the questionnaire (which allows important feedback for the stakeholders) represents only a first scientific step. The subsequent steps that this study aims to stimulate are the integration of this questionnaire together with other solutions during the application of agreement initiatives. The Consensus Conferences [71,72,73], for example, could be an important agreement initiative and could certainly benefit (in the context of the activities of the working groups [74,75,76]) from the use of electronic questionnaires that provide for structured feedback and virtual focus groups.

Our questionnaire has the above-listed limits. However, it *has the merit* of having initiated this approach, in a delicate issue (*medical robotics*), and of being a stimulus for the *scientific societies involved*. It is in line with other similar initiatives in the *health domain*. International scientific meetings, promoted *by scientific societies* [77], now include sections dedicated to the problems of *Cyb* in the HCI. In a study [78], just presented in [77], the importance of using dedicated surveys is stressed, to improve understanding of behaviors at risk, as regards *Cyb, when using HCI* in the *health domain*. Our study is in this direction. Likewise, it addresses the *Cyb* problems in a new field of the HCI, the human robot interaction (a complex HCI with mechatronics) [79], through a wide-ranging investigation, using a questionnaire and involving concerned actors..

## 5. Conclusions

Rehabilitation and assistance robots represent an opportunity for the health domain [7,8]. The use of these robots has important implications. They can be used with fragile patients or people with disabilities, in rehabilitation and assistance processes. They can be used in psychological and cognitive rehabilitation processes for children and other subjects with communication disabilities, as in the case of SRs. Therefore, their use can have important physical and psychological implications. Furthermore, the software in these devices interact with sensible data. Cybersecurity has therefore become an important issue to face, starting from the insiders. We have proposed an investigation based on a questionnaire submitted to physiotherapists. The investigation showed the following highlights:

− The questionnaire, dedicated to physiotherapists and with reference to *CybH* in robotics, has the advantage of allowing a monitoring of roles and interactions in the workplace, a monitoring of training received, a self-assessment of risks, and a virtual focus group.
− The questions enabled us to collect important data on: (a) the use of robotics and the direct involvement in *CybH*; (b) training in robotics, cybersecurity, and other disciplines; (c) the self-perception of *Cyb* and robotics; (d) opinions, suggestions, and experiences.
− The data concerned both subjects with only training experiences and subjects with direct work experience.
− At the time of the survey, 102 (32.27%) respondents used rehabilitation robotics in the workplace. All have highlighted their role as user, but only 29 (9.18%) had a direct involvement with *Cyb*. Only 5 respondents stated that they were dealing with SRs. Of these, 3 (0.95%) were observers and 2 (0.63%) were users, while only one (0.32%) had a direct involvement in *Cyb*.
− An acceptable training regarding robotics and other related training modules. An unacceptable training when dealing, in detail, with *Cyb* issues. A training that highlighted gaps for the regulation issues on *Cyb* (also referred to robotics). An awareness, during the training, on the involvement of the physiotherapist in *Cyb* (also related to robotics).
− The possibility for physiotherapists to self-assess themselves in some *Cyb* scenarios proposed in respect of robots.
− Opinions on emerging risks and wishes in this field (as, for example, to continue the use of the questionnaire and to create specific working groups). Both the participants with a direct experience of robotics and participants with only a training experience narrated experiences in this field with enthusiasm: 302 (95.57%) described their experiences with robotics, categorized after data mining, showing that 55 reported *Cyb* problems with robotics in the *workplace*. This, very importantly, highlighted that 52.3% of the physiotherapists engaged with robotics in the workplace reported a *Cyb* problem. The most frequent incidents were *denial of service* (7), which involved a network with LOLI, UPLI, BA, and ransomware attacks on the data of a LOLI platform (5).

## 6. Future Work

The needs for future work that emerge from this study concern both continuation in the field of rehabilitation and assistance robotics and the activation of similar initiatives in other sectors of robotics.

### 6.1. Future Initiatives in the Field of Rehabilitation and Assistance Robotics

Future developments of this study are foreseen to include:

- An improvement of the electronic questionnaire, with a standardization of the same, interacting with the scientific societies;
- Using it for specific periodic monitoring and investigations;
- Stimulating the stakeholders for the creation of multidisciplinary workgroups to address *Cyb* (ranging from engineering to machine ethics, legal and policy issues);
- Expansion to other professional groups.

### 6.2. Suggestions for Future Developments in Other Sectors of Medical Robotics

The Policy Department for Economic, Scientific and Quality of Life Policies, of the European Parliament, identified the most interesting applications for the medical robots [79]: *Robotic surgery, care and socially assistive robots, rehabilitation systems, training for health and care workers*. The sector is wide, complex and with numerous implications for CybH. What emerged in this study may be a stimulus for those engaged in other areas of medical robotics to initiate similar studies focused on *Cyb*.

## Figures and Tables

**Table 1:** Characteristics of the participants.

Submission	Participants	Males/Females	Min Age/Max Age	Mean Age
Physiotherapists	316	162/154	23/58	38.47

**Table 2:** Sections of the questionnaire.

Section	Title
***Section 1***	Demographic data
***Section 2***	Robotics and cybersecurity in the workplace
***Section 3***	Training in cybersecurity and robotics
***Section 4***	Self-assessment on cybersecurity and robotics
***Section 5***	Proposals and collection of personal cases of cyber-risk

**Table 3:** Perceived degree of training on SRs, robots for BA, robots for LOLI, robots for UPLI.

Question	Mean	CI 95%
Upper limb rehabilitation	4.55	±0.38
Lower limb rehabilitation	4.43	±0.37
Balance	4.42	±0.38
Social robot	3.63	±0.38

**Table 4:** Perceived degree of training on informatics, mHealth, eHealth, cybersecurity.

Question	Mean	CI 95%
Informatics	4.57	±0.38
Electronic health	4.41	±0.37
Mobile health	4.45	±0.37
Cybersecurity	2.49	±0.36

**Table 5:** Assessed knowledge on cybersecurity.

Question	Mean	CI 95%
Malware (virus, Trojan, ransomware, scareware...)	3.57	±0.36
Man in the middle	2.41	±0.37
Denial of service (DoS)	2.45	±0.38
Distributed denial of service (DDoS)	2.49	±0.35
Spoofing	2.46	±0.38
Sniffing	3.13	±0.37
Phishing	3.60	±0.38
Data breach	2.46	±0.37
Back door	2.46	±0.33
Password cracker	3.56	±0.32

**Table 6:** Perception on the influence of Cyb in Robotics.

Question	Mean	CI 95%
SR	4.58	±0.38
BA	3.87	±0.37
UPLI	4.21	±0.37
LOLI	4.22	±0.36

**Table 7:** Results relating to the graded questions with the details of the assessment.

Question	BA	LOLI	UPLI	SR
Malware (virus, Trojan, ransomware, scareware...)	310	309	311	308
Man in the middle	251	247	259	253
Denial of service (DoS)	252	261	249	248
Distributed denial of service (DDoS)	249	252	253	250
Spoofing	247	250	252	249
Sniffing	278	281	293	300
Phishing	309	310	310	312
Data breach	279	268	269	288
Back door	269	257	258	267
Password cracker	309	309	3011	312

**Table 8:** Results relating to the graded questions with the details of the assessment.

	BA	LOLI	UPLI	SR
Physical damage	308	309	307	212
Physical harm	307	306	305	213
Psychological harm	13	14	18	309

**Table 9:** Level of awareness in cyber-risk scenarios for UPLI.

Level of Awareness	Mean	CI 95%
During software update process	3.89	±0.37
During upload process	3.92	±0.38
General vulnerability	3.63	±0.37
On the possible effect on the patient/practitioner’s health and safety	4.39	±0.33

**Table 10:** Level of awareness in cyber-risk scenarios for LOLI.

Level of Awareness	Mean	CI 95%
During software update process	3.88	±0.37
During upload process	3.99	±0.38
General vulnerability	3.64	±0.37
On the possible effect on the patient/practitioner’s health and safety	4.39	±0.33

**Table 11:** Level of awareness in cyber-risk scenarios for BA.

Level of Awareness	Mean	CI 95%
During software update process	3.89	±0.37
During upload process	3.95	±0.38
General vulnerability	3.57	±0.37
On the possible effect on the patient/practitioner’s health and safety	4.41	±0.33

**Table 12:** Level of awareness in cyber-risk scenarios for SR.

Level of Awareness	Mean	CI 95%
During software update process	3.47	±0.39
During upload process	3.44	±0.43
General vulnerability	3.45	±0.41
On the possible effect on the patient/practitioner’s health and safety	4.28	±0.41

**Table 13:** Suggestions on the cyber risk to face.

Priority	Suggestion	Number of Suggestions
1	Risk of physical damage for incorrect imposition of kinematic/dynamic therapy	89
2	Risk of incorrect recording of the trials	72
3	Risk of out-of-control behavior of the SR	16

**Table 14:** Suggestions on the actions to consider.

Priority	Suggestion	Number of Suggestions
1	Launch periodic monitoring actions led by scientific societies.	83
2	Create heterogeneous national working groups to address cybersecurity in the 4 sectors of robotics	46
3	Launch training initiatives on the various issues of cybersecurity applied to the various sectors of robotics.	26

## Data Availability

Not applicable.
